# Amyloid β-Exposed Human Astrocytes Overproduce Phospho-Tau and Overrelease It within Exosomes, Effects Suppressed by Calcilytic NPS 2143—Further Implications for Alzheimer's Therapy

**DOI:** 10.3389/fnins.2017.00217

**Published:** 2017-04-20

**Authors:** Anna Chiarini, Ubaldo Armato, Emanuela Gardenal, Li Gui, Ilaria Dal Prà

**Affiliations:** ^1^Human Histology and Embryology Unit, Medical School, University of VeronaVerona, Venetia, Italy; ^2^Department of Neurology, Southwest Hospital, Third Military Medical UniversityChongqing, China

**Keywords:** amyloid-β, Tau, adult human astrocytes, exosome, calcium-sensing receptor, calcilytics, Alzheimer's disease, GSK-3β

## Abstract

The two main drivers of Alzheimer's disease (AD), amyloid-β (Aβ) and hyperphosphorylated Tau (p-Tau) oligomers, cooperatively accelerate AD progression, but a hot debate is still ongoing about which of the two appears first. Here we present preliminary evidence showing that Tau and p-Tau are expressed by untransformed cortical adult human astrocytes in culture and that exposure of such cells to an Aβ_42_ proxy, Aβ_25−35_, which binds the calcium-sensing receptor (CaSR) and activates its signaling, significantly increases intracellular p-Tau levels, an effect CaSR antagonist (calcilytic) NPS 2143 wholly hinders. The astrocytes also release both Tau and p-Tau by means of exosomes into the extracellular medium, an activity that could mediate p-Tau diffusion within the brain. Preliminary data also indicate that exosomal levels of p-Tau increase after Aβ_25−35_ exposure, but remain unchanged in cells pre-treated for 30-min with NPS 2143 before adding Aβ_25−35_. Thus, our previous and present findings raise the unifying prospect that Aβ•CaSR signaling plays a crucial role in AD development and progression by simultaneously activating (i) the amyloidogenic processing of amyloid precursor holoprotein, whose upshot is a surplus production and secretion of Aβ_42_ oligomers, and (ii) the GSK-3β-mediated increased production of p-Tau oligomers which are next released extracellularly inside exosomes. Therefore, as calcilytics suppress both effects on Aβ_42_ and p-Tau metabolic handling, these highly selective antagonists of pathological Aβ•CaSR signaling would effectively halt AD's progressive spread preserving patients' cognition and life quality.

## Introduction

Late onset (non-familial) *Alzheimer's disease* (LOAD) is the most common dementia afflicting millions of people worldwide. It is characterized by extracellular deposits of fibrillar Aβ_42_ peptides (neuritic or senile plaques) and by intracellular pre-tangles and neurofibrillary tangles (NFTs) of phosphorylated Tau (p-Tau) protein (Selkoe, 2008a,b; Grinberg et al., 2009; Braak et al., 2011; Attems et al., 2012; Elobeid et al., 2012; Braak and Del Tredici, 2013). LOAD neuropathology develops stealthily during 20–40 years before its clinical emergence (Masdeu et al., 2012). It is thought to be driven by the tandem toxic activities of oligomers of amyloid β (Aβ-os) and p-Tau (p-Tau-os) let out from affected cell processes via exocytosis and/or exosomes (or extracellular vesicles) (Saman et al., 2012). Both released Aβ-os and p-Tau-os thus reach adjacent or connected cells, inducing them to release in their turn *newly* produced Aβ-os and p-Tau-os. Thus, LOAD spreads from entorhinal cortex layer II to upper cognitive cortical areas killing unreplaceable neurons and disconnecting their networks in its path (Morrison and Hof, 1997; Selkoe, 2008a,b; Khan et al., 2014). Notably, p-Tau can be neurotoxic all by itself too in advanced AD and in *tauopathies* caused by mechanisms independent of Aβ-os or senile plaques (Medeiros et al., 2013). Which of the two main AD toxic drivers appears first is controversial. According to some, a very early surfacing and spread of intraneuronal p-Tau pathology (i.e., pre-tangles, NFTs, and neuropil threads) from the brainstem to the cerebral cortex occurs in the total absence of extra-neuronal Aβ_42_ accumulation (Braak et al., 2013; see also below). However, others hold that poorly detectable soluble Aβ_42_-os are the earliest LOAD drivers (Selkoe, 2008a,b; Crimins et al., 2013; Kayed and Lasagna-Reeves, 2013), bringing about p-Tau-os, NFTs, and synaptic pathology in the total absence of senile plaques (reviewed by Klein, 2013). Indeed, the para-hippocampal and inferior temporal *gyri* of 8-year-old Down's syndrome children already exhibited Aβ deposits (Leverenz and Raskind, 1998). In fact, they had a chromosome 21 tri-ploidy and three copies of the Aβ precursor holoprotein (hAPP) gene which made them susceptible to develop an early AD neuropathology. In long-term *in vitro* three-dimensional cultures of neural cells, Aβ-os build-up preceded any p-Tau-os detection further strengthening the view Aβ-os are the first AD drivers (Choi et al., 2014) while also stressing the usefulness of preclinical *in vitro* models to elucidate molecular mechanisms underlying AD development.

Accordingly, p-Tau-os seem to occupy the second tier in the hierarchy of AD drivers (Clavaguera et al., 2009, 2013a,b; Gerson and Kayed, 2013). Under physiological conditions, Tau is a soluble microtubule-associated phosphoprotein (MAP) strongly expressed in neurons (Goedert, 1993) and human astrocytes (Ferrer et al., 2002; Tanji et al., 2003; Wakabayashi et al., 2006, and present results). Tau moiety encompasses a microtubule-binding C-terminal repeat domain, a central proline-rich domain, and an N-terminal domain interacting with membranes and/or other proteins. In human adult brain, an alternatively spliced single gene allows the expression of six Tau isoforms, of which 4RTau and 3RTau are the most intensely produced and phosphorylated ones (Hanger et al., 1998; Hasegawa, 2006). Soluble Tau monomers are physiologically gathered within neurons' axons where they tightly bind, stabilize, and help elongate microtubules, besides associating with the plasma membrane (Pooler and Hanger, 2010). They partake in the fast anterograde transport (FAT) of various cargos (e.g., mitochondria, synaptic vesicles) on kinesin motors linked to microtubule trackways. Tau is rapidly and reversibly phosphorylated by several protein kinases and phosphatases. Soluble Tau purified from normal human brains is phosphorylated at about 10 sites only (Hanger et al., 2007; Sergeant et al., 2008). Yet, Tau is endowed with 85 serine and threonine phosphorylable sites, and glycogen synthase kinase (GSK)-3β is the main kinase for 45 of them in poorly soluble p-Tau (Buée et al., 2000; Sergeant et al., 2008; Tavares et al., 2013). When GSK-3β hyper-phosphorylates Tau, the latter's ability to promote normal microtubule assembly wanes (Utton et al., 1997). Then p-Tau detaches from tubulin, destabilizing and disassembling microtubules (Lindwall and Cole, 1984; Drechsel et al., 1992). Hence, increases in p-Tau due to GSK-3β activity surges are typical marks of blunted physiological functions (e.g., axonal transport, etc.) in neurons (LaPointe et al., 2009). In AD and various tauopathies, p-Tau accumulates intracellularly as filaments, pre-tangles, and insoluble NFTs, and hyper-reacts to anti-p-Tau-specific antibodies (Greenberg and Davies, 1990; Ballatore et al., 2007; Gendron and Petrucelli, 2009). Not surprisingly, GSK-3β colocalizes with NFTs in AD and AD-related disorders (Hanger et al., 1998; Ferrer et al., 2002; Hanger and Noble, 2011). Notably, p-Tau from AD brains coimmunoprecipitates with a fraction of Tau, revealing that AD's p-Tau-os are Tau/p-Tau mixtures (Köpke et al., 1993) just as AD's Tau filaments or fibrils are (Alonso et al., 1997). The pathological role of GSK-3β-phosphorylated Tau is supported by results in mouse transgenic AD or tauopathy models, in which GSK-3β inhibition lessened Tau phosphorylation and aggregation and axonal degeneration (Serenó et al., 2009; Leroy et al., 2010). And, SB-415286, a specific inhibitor of GSK-3β activity, decreased p-Tau levels and kept cultured primary neurons viable (Gross et al., 2001).

According to Braak et al. (2011) and Braak and Del Tredici (2012, 2013), abnormal p-Tau-os in non-fibrillar form were seen within proximal axons and AT-8 antibody-positive pre-tangles were observed within the somata and dendrites of projection neurons of brainstem *locus coeruleus*/ *subcoeruleus* of young boys well before they became manifest in the hippocampal trans-entorhinal region, the putative site of AD onset (Khan et al., 2014). The authors posited AD begins from brainstem neurons which inject neurotoxic p-Tau-os into higher cortical regions (Hertz, 1989; Agnati et al., 1995)—a process starting AD's “*Braak stages*” (Braak et al., 2011; Braak and Del Tredici, 2012, 2013). Concurrently, others set forth the concept of trans-synaptically transmittable, prion-like, soluble Tau-os which by destroying first synapses, then axons, and finally neurons would disconnect neuronal networks (Clavaguera et al., 2009, 2013a,b; Lasagna-Reeves et al., 2012; de Calignon et al., 2012; Gerson and Kayed, 2013). However, p-Tau-os cannot cross synaptic terminals as prions do (Stranahan and Mattson, 2010).

Hitherto, as with Aβs, neurons were held as the main source of Tau/p-Tau (Wu et al., 2013; Avila et al., 2014). But what about other neural cell types? Wakabayashi et al. (2006) reported the co-localization of Aβ and p-Tau in the *subiculum* and entorhinal cortex astrocytes of a patient with corticobasal degeneration. They interpreted this finding as follows: “*the phagocytosis of A*β *coincides with production of phospho-Tau in the same reactive astrocytes*.” However, as we previously showed, untreated cortical untransformed adult human astrocytes produce basal amounts of Aβ_42_ and, once challenged with exogenous fibrillar (f)Aβ_25−35_, an Aβ_42_ proxy, make and release significantly greater amounts of endogenous Aβ_42_/Aβ_42_-os (Armato et al., 2013; Dal Prà et al., 2015; Chiarini et al., 2016). We also demonstrated exogenous fAβ_25−35_-os bind the astrocytes' and neurons' calcium-sensing receptors (CaSRs) (Dal Prà et al., 2014a,b) and activate their signaling pathways heightening the production and secretion of endogenous Aβ_42_/Aβ_42_-os. In fact, a specific CaSR *agonist* (calcimimetic), NPS R-568 (Nemeth and Goodman, 2016), mimicked the enhancing effect of exogenous fAβ_25−35_-os on Aβ_42_/Aβ_42_-os secretion (Armato et al., 2013). Conversely, a highly selective CaSR *antagonist* (calcilytic), NPS 2143 (Nemeth and Goodman, 2016), fully quelled the fAβ_25−35_-os-induced surplus *de novo* production and secretion of Aβ_42_/Aβ_42_-os in both human neurons and astrocytes (Armato et al., 2013; Dal Prà et al., 2015; Chiarini et al., 2016). Interestingly, human MIC neuroblasts secrete Tau enclosed within exosomes which are found in human cerebrospinal fluid too (Saman et al., 2012). Even plasma astrocyte-derived exosomes contain p-Tau proteins (Goetzl et al., 2016). And, neurons uptake exogenous Tau proteins via endocytosis into the somatodentritic compartments or axon termini from which they are conveyed to various cell sites (Wu et al., 2013). Altogether, these data indicated the urgent need to reassess the relationship between Aβ peptides exposure and p-Tau production and release in adult human astrocytes and neurons. Therefore, we undertook a pilot study using as model cultured human astrocytes (Armato et al., 2013) whose preliminary results we herein report. Details on materials and methods we used are in Supplementary Materials.

## Results

### Aβ•CaSR signaling increases GSK-3β Tau kinase activity in normal adult human astrocytes

**Figure 1:**
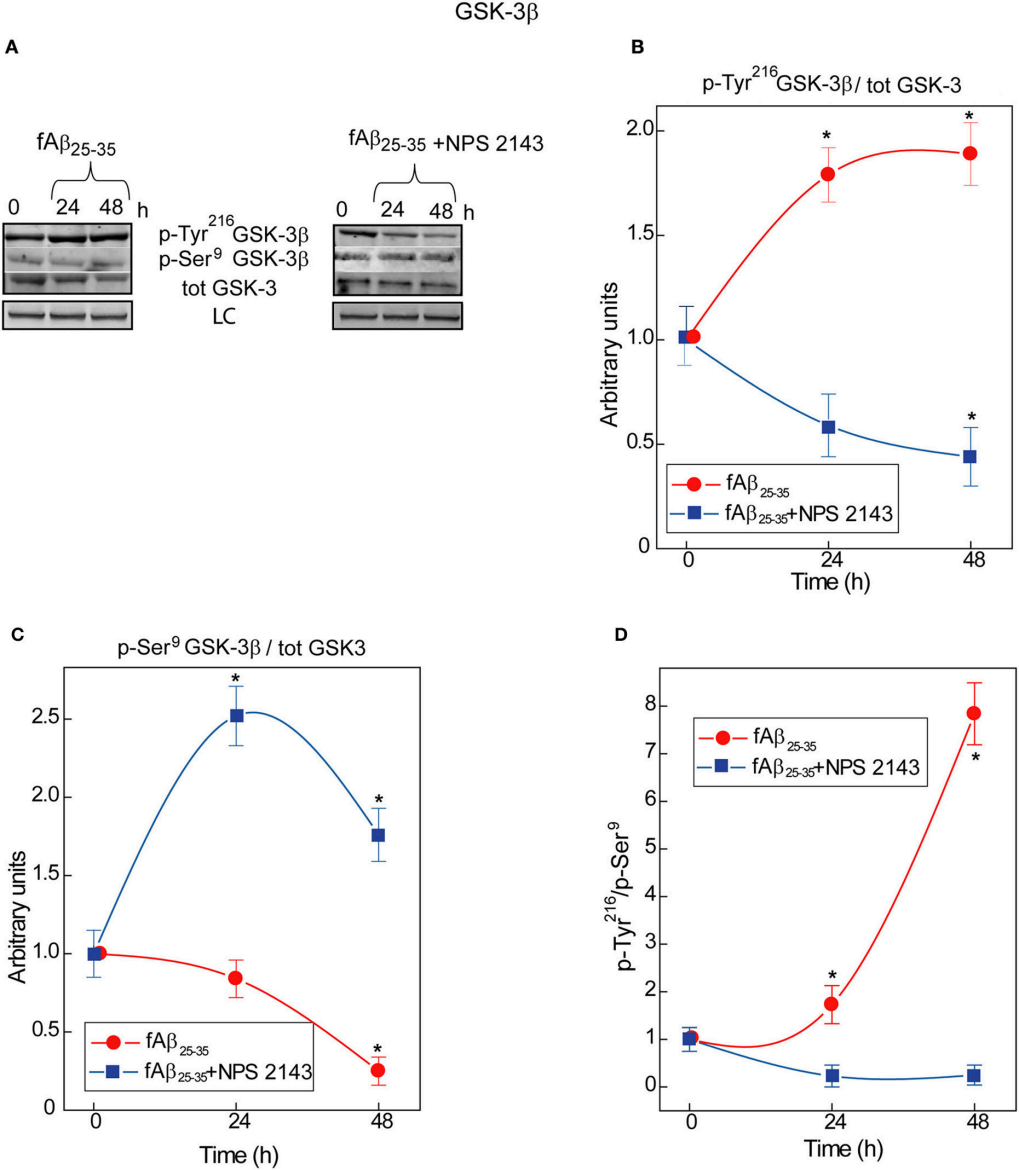
**Time course of GSK-3β phosphorylations in human adult astrocytes exposed to fAβ_**25−35**_ ±NPS 2143. (A)** Typical immunoblots of human astrocytes total protein lysates illustrating the changes in the specific bands corresponding to p-Tyr^216^GSK-3β, p-Ser^9^GSK-3β, and total (*tot*) GSK-3 total in untreated (control) cells and in cells exposed to fAβ_25−35_± a short (30-min) treatment with calcilytic NPS 2143. LC, loading control (lamin B1). **(B)** p-Tyr^216^GSK-3β/GSK-3 ratio increased in fAβ_25−35_ (20 μM)-treated cells (*red line*), an effect a short NPS 2143 pre-treatment completely prevented (*blue line*). **(C)** p-Ser^9^GSK-3β/GSK-3 ratio decreased under the stimulus of fAβ_25−35_ alone whereas it increased when of fAβ_25−35_ administration was preceded by a 30-min pre-treatment with calcilytic NPS 2143 (*blue line*). **(D)** As indicated by the augmented pTyr^216^GSK-3β/pSer^9^GSK-3β ratio, the activity of GSK-3β hugely increased in the astrocytes exposed to exogenous fAβ_25−35_ alone (*red line*), but was significantly downregulated when calcilytic NPS 2143 was given for 30-min before fAβ_25−35_ to the astrocyte cultures (*blue line*). Points in the curves express the mean ratios between the specific phosphorylated sites and total GSK-3 ± SEMs from 3 distinct experiments. ^*^P < 0.01 vs. control (0-h) values.

An ongoing balance between phosphorylation and de-phosphorylation of some of its serine (Ser) and tyrosine (Tyr) residues controls GSK-3β enzymatic activity: relative increases in Tyr^216^ phosphorylation upregulate and, conversely, of Ser^9^ phosphorylation downregulate it (Forde and Dale, 2007) heightening or reducing, respectively, p-Tau levels (Qian et al., 2010). In human adult astrocyte lysates an exposure to exogenous fAβ_25−35_ nearly doubles between 0 and 48-h the p-Tyr^216^GSK-3β/total GSK-3 ratio values (Figures 1A,B) while simultaneously curtailing p-Ser^9^GSK-3β/total GSK-3 ratio values (Figures 1A,C). As a consequence, the p-Tyr^216^/p-Ser^9^ ratio values and hence GSK-3β activity increase up to 8-fold in fAβ_25−35_-exposed astrocytes (Figure 1D) as the latter does in hippocampal neurons (Takashima et al., 1998). Remarkably, a 30 min pre-treatment with calcilytic NPS 2143 totally quells the raise in fAβ_25−35_-induced p-Tyr^216^GSK-3β levels; contrariwise, the p-Tyr^216^GSK-3β/total GSK-3 ratio values fall below control values (Figures 1A,B). Concurrently, NPS 2143 increases the p-Ser^9^GSK-3β/total GSK-3 ratio values well above control ones (Figures 1A,C). As a result, the p-Tyr^216^/p-Ser^9^ ratio values and hence activity levels fall below basal values (Figure 1D). These results constitute the first evidence that pathological Aβ•CaSR signaling directly intensifies GSK-3β activity besides rising endogenous Aβ_42_/Aβ_42_-os production/release from the cortical adult human astrocytes.

### Expression of Tau protein isoforms in adult human astrocytes

**Figure 2:**
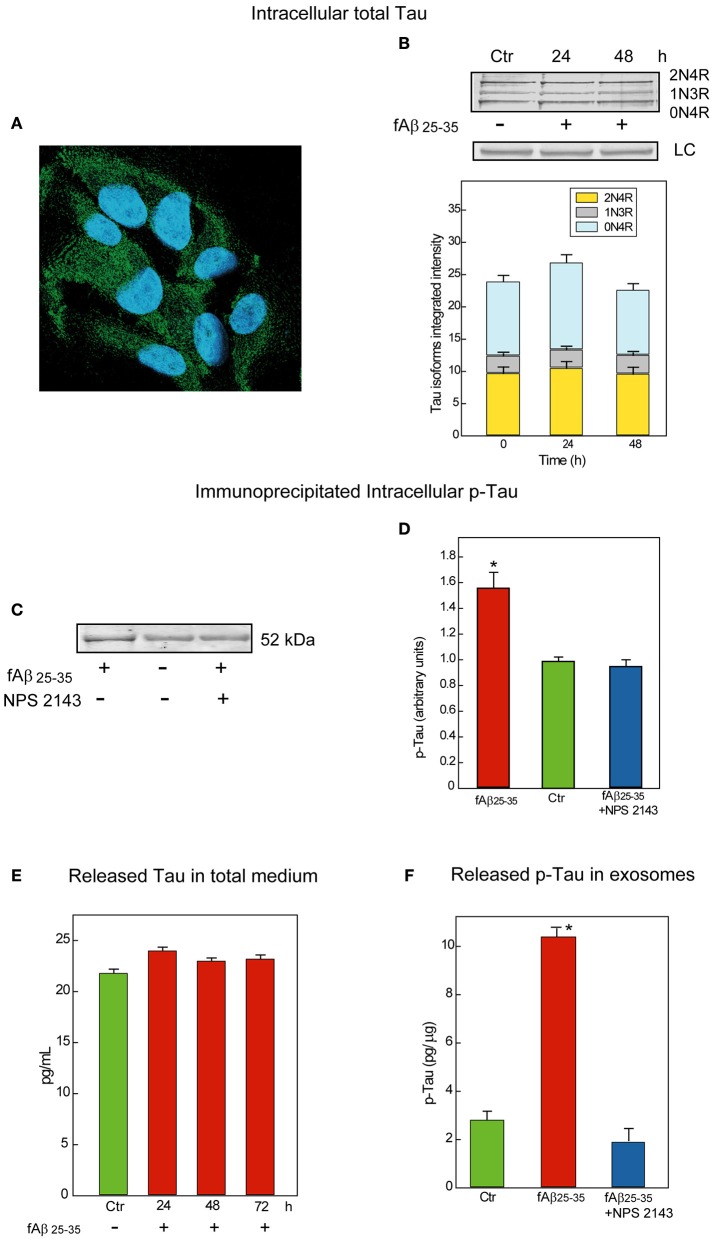
**Characterization and release of Tau/p-Tau from human adult astrocytes. (A)** Immunofluorescence staining of total Tau (antibody HT7) in untreated astrocytes as a diffuse granular green labeling of the cytoplasms. Nuclei are stained with DAPI. Merged picture. Magnification, 640X. **(B)** Top: Typical immunoblot analysis of the Tau isoforms astrocytes express when untreated (*Ctr*) or exposed for 24 or 48-h to fAβ_25−35_ alone treatment. Notably, 1N3R, 0N4R, and 2N4R are known as the Tau isoforms involved in the formation of pre-tangles and NFTs (Espinoza et al., 2008). LC, load controls (lamin B1). Bottom: Densitometric assessment of the three Tau isoforms integrated intensities. No significant changes are detectable (*n* = 3). **(C)** Typical immunoblot analysis of immunoprecipitated p-Tau in lysates from untreated and 48-h fAβ_25−35_±NPS 2143-treated astrocytes. Adding NPS 2143 pretreatment totally blocks any increase in p-Tau levels elicited fAβ_25−35_ alone which remain at basal values. **(D)** Densitometric data corresponding to p-Tau specific bands are shown as bars which are the means ± SEMs expressed as arbitrary units (*n* = 3), and normalized taking as 1.0 the values of untreated astrocytes. ^*^P < 0.001 vs. control values. **(E)** Time course of Tau protein release in the growth medium of un-treated and fAβ_25−35_-exposed astrocytes. The ELISA values of total Tau protein detected in 24 to 72-h astrocytes treated with fAβ fAβ_25−35_ do not significantly differ from control ones at each time point examined. Bars are the mean values ± SEMs of three experiments in duplicate. **(F)** p-Tau is released in exosomes under physiological conditions and its amount remarkably increases in fAβ_25−35_ treated astrocytes, but adding NPS 2143 for 30 min prior to exposing astrocytes to fAβ_25−35_ prevents any increase in exosomal p-Tau from occurring. Bars are means ± SEMs of three experiments in duplicate. ^*^P < 0.001 vs. control values and vs. fAβ_25−35_±NPS 2143-treated values.

First, we examined Tau proteins expression in astrocytes by means of immunofluorescence staining and observed a diffuse granular Tau-immunoreactivity pattern mostly in the cytoplasm (Figure 2A). Cytoplasmic granular Tau aggregates were previously reported (Ward et al., 2013). Then, to identify the several Tau isoforms involved, we analyzed via Western blotting whole astrocyte lysates from both untreated and fAβ_25−35_-treated cells (Figure 2B). We used a *pan*-Tau antibody which recognizes all Tau isoforms (Tran et al., 2011), and confirmed the identity of each specific isoform band by using a commercially available Tau protein ladder composed of the six known Tau isoforms. Thus, in both untreated and fAβ_25−35_-treated astrocytes, the *pan*-Tau antibody recognized three resolvable Tau bands in the size range between 45 and 60 kDa, corresponding to Tau isoforms 2N4R, 1N3R, and 0N4R, which are those involved in the formation of pre-tangles and NFTs (Espinoza et al., 2008). Total Tau levels were alike in untreated and fAβ_25−35_-treated cultures, suggesting no changes in total Tau isoforms expression were elicited by fAβ_25−35_-exposure vs. no treatment in the astrocytes at least during 72-h of treatment.

### fAβ_25−35_-treated astrocytes have increased intracellular p-Tau levels NPS 2143 suppresses

Via Kinex Antibody Microarray™ we analyzed the p-Tau pattern in total cellular lysates of untreated and fAβ_25−35_-treated astrocytes. Using phospho-site-specific antibodies this analysis tracked the main Tau phospho-sites regulated by GSK-3β activity (Hanger and Noble, 2011) and demonstrated that the phosphorylation levels of Ser^199^, Ser^396^, and Ser^422^ of the Tau molecule were remarkably increased in 24-h fAβ_25−35−_exposed astrocytes (not shown). This preliminary evaluation invited further investigations in order to specifically establish the amount of increased p-Tau proteins.

Therefore, we first immunoprecipitated the *phosphorylated* proteins from whole lysates of untreated and fAβ_25−35_-exposed astrocytes—the latter pre-treated or not pre-treated with calcilytic NPS 2143 since, as we just saw, Aβ•CaSR-signaling regulates GSK-3β activity (Figure 1). Next, we probed the immunoblots of the immunoprecipitated total phospho-proteins with a specific anti-Tau antibody. As shown in Figure 2C, the levels of p-Tau markedly increased in the fAβ_25−35_-treated astrocytes as compared to untreated cells. Importantly, NPS 2143 pre-treatment kept p-Tau at physiological (untreated control) levels in the fAβ_25−35_-exposed cells (Figures 2C,D).

### Exosome-associated Tau and p-Tau releases from human adult astrocytes

Untreated and Aβ-exposed cortical adult human astrocytes also release Tau proteins into the growth medium. In preliminary experiments, by using an ELISA assay with a sensitivity <10 pg/mL we found total Tau protein levels of 21.8 pg/mL in 72-h untreated (control) astrocytes medium samples. In the medium of 24–72-h fAβ_25−35_-treated astrocytes, we detected unchanging values of the total Tau proteins which did not differ from control ones, suggesting the operation of a steady balance between Tau release and Tau re-uptake (Figure 2E).

But, is Tau/p-Tau secreted free into the growth medium or is it enclosed within exosomes? To answer this question, we started analyzing Tau release under physiological conditions. We purified exosomes from media conditioned for 72-h by untreated astrocytes and then quantified Tau by means of a specific ELISA kit in exosome fractions purified from them and in exosome-depleted media samples. This analysis showed Tau proteins associated with the exosome fractions and the exosomal Tau levels did not significantly differ from those found in whole media samples (~24.3 pg/mL). Conversely, under the same conditions, Tau could not be detected at all in exosome-depleted media samples. Therefore, all the Tau human astrocytes release is enclosed within exosomes.

Next, we investigated whether endogenous Tau released from astrocytes within exosomes was phosphorylated. By means of a p-Tau-specific ELISA kit we could demonstrate that under physiological conditions p-Tau secretion occurred inside exosomes too (Figure 2F, *Ctr*). Finally, we asked whether an exposure to fAβs_25−35_ ±NPS 2143 affected the amount of p-Tau released via exosomes. Our pilot results (*n* = 3) hint that this is indeed the case. In fact, using the same p-Tau-specific ELISA kit we observed that exosome-associated p-Tau increased markedly with fAβ_25−35_-treated astrocytes as compared to untreated ones, but a 30-min pretreatment with NPS 2143 of the fAβ_25−35_-exposed cells wholly quelled any exosomal p-Tau surge keeping it at controls' levels (Figure 2F). Further in depth studies will validate and extend these pilot findings.

## Conclusions and future perspectives

AD is a complex human illness which is only partially modeled in rodents. Using as paradigm cultured cortical untransformed adult human astrocytes, which differ from rodents' ones from both morphological and functional standpoints (Ogata and Kosaka, 2002; Tsai et al., 2012; Robertson, 2014) and are not killed by accumulating Aβs (Armato et al., 2013; Dal Prà et al., 2015; Chiarini et al., 2016) has brought to light molecular mechanisms which likely partake in AD's onset and progression. Previous work showed exogenous Aβs bind the plasma membrane CaSRs of human astrocytes and neurons (Dal Prà et al., 2014a,b). The thus triggered pathological Aβ•CaSR signaling increases the amyloidogenic processing of hAPP which entails a surplus extracellular secretion of endogenous Aβ_42_ from both cell types (Armato et al., 2013; Dal Prà et al., 2015; Chiarini et al., 2016). Additionally, Aβ•CaSR signaling elicits neurotoxic surpluses of nitric oxide and VEGF-A production and release from human astrocytes (Dal Prà et al., 2005, 2014b; Armato et al., 2013). Under these multiple neurotoxic insults human cortical neurons start progressively dying (Armato et al., 2013; Chiarini et al., 2015). And the Aβ_42_-os accumulating in the neuropil spread to bind and activate the CaSRs of adjacent neurons and astrocytes, thus promoting further Aβ_42_-os production and diffusion (Dal Prà et al., 2015; Chiarini et al., 2016). Remarkably, a highly selective CaSR antagonist (calcilytic), NPS 2143, effectively blocks the Aβ•CaSR signaling and all of its neurotoxic consequences, preserving human neurons' viability notwithstanding a persisting Aβ-os presence. Thus, from the Aβs standpoint calcilytics would be effective as anti-AD therapeutics (Armato et al., 2013; Dal Prà et al., 2014b, 2015; Chiarini et al., 2015, 2016).

However, we cannot ignore the main drivers of AD are both Aβ-os and p-Tau-os. It has been argued that p-Tau-os advent precedes Aβ_42_-os' (Braak et al., 2011; Elobeid et al., 2012). But, evidence also exists that Aβ_42_-os manifestation antecedes p-Tau-os' (Leverenz and Raskind, 1998; Klein, 2013; Choi et al., 2014). Beyond question is only that when both Aβ-os and p-Tau-os are present, AD course toward patient's demise briskly accelerates (Ittner and Gotz, 2011). So how this drivers' antinomy might be solved? Our findings show that besides stimulating the pathological amyloidogenic processing of hAPP into Aβ_42_, Aβ•CaSR signaling increases the activity of GSK-3β and hence the intracellular accumulation of p-Tau in human astrocytes. Next, mixtures of both Tau and p-Tau are enclosed within exosomes and released into the extracellular environment. In the static *in vitro* system we used, a balance is kept between release and reuptake of Tau/p-Tau-containing exosomes. *In vivo*, such Tau/p-Tau-containing exosomes would spread into the neuropil to be uptaken by adjacent neurons and astrocytes. Given astrocytes' higher numbers, a persistent Aβ-elicited exosomal p-Tau overrelease would exacerbate the neurons' toxic accumulation of p-Taues favoring their aggregation into pre-tangles and NFTs.

Therefore, our present results raise the enticing prospect that pathological Aβ•CaSR signaling would simultaneously trigger both the Aβ-mediated and the p-Tau-mediated neurotoxic mechanisms driving AD neuropathology. The other exciting facet of these findings is that calcilytic NPS 2143 can fully suppress all the neurotoxic effects Aβ•CaSR signaling wakes up, including the intracellular accumulation and exosomal release of p-Tau surpluses from human astrocytes. Further work will assess whether calcilytics similarly hinder excess p-Tau production/release from Aβ-exposed human cortical neurons. However, NPS 2143 does suppress the Aβ_42_ surplus production and secretion from Aβ-exposed human neurons (Armato et al., 2013). Therefore, it seems feasible that NPS 2143 would block neurons' GSK-3β's Tau hyperphosphorylating activity too.

In conclusion, with all the advisable caution our preclinical findings deserve, the present perspective suggests CaSR antagonists would block the intracerebral seeding of both AD main drivers, the Aβs and p-Taues, besides accessory neurotoxic factors like NO and VEGF-A surpluses. Accordingly, if administered early enough, calcilytics would freeze AD progression and preserve patients' ongoing cognitive abilities and quality of life.

## Ethics statement

This research work was approved by the Ethical Committee of the Integrated Verona University-Hospital Co., Prog. No. CE118CESC.

## Author contributions

AC, UA, and IDP conceived the research and designed the experiments. AC, IDP, and EG performed the experiments and collected the results. UA and LG statistically analyzed the data. AC, UA, and IDP interpreted the results. The manuscript was principally written and revised by UA, AC, and IDP. All the authors critically reviewed the manuscript for important intellectual content and approved the final submitted manuscript.

## Funding

This work was supported in part by the Italian Ministry for University and Research (F.U.R. 2014 and 2015 allotments to AC, and IDP).

### Conflict of interest statement

The authors declare that the research was conducted in the absence of any commercial or financial relationships that could be construed as a potential conflict of interest.
