# Supplementary material for: Amyloid β-Exposed Human Astrocytes Overproduce Phospho-Tau and Overrelease It within Exosomes, Effects Suppressed by Calcilytic NPS 2143—Further Implications for Alzheimer's Therapy
Source: Front Neurosci. 2017 Apr 20;11:217. doi: 10.3389/fnins.2017.00217 (PMC5397492; doi:10.3389/fnins.2017.00217)
Supplement: Supplementary file 1 [file DataSheet1.docx]

**SUPPLEMENTARY MATERIALS**

**Amyloid β-exposed human astrocytes overproduce phospho-Tau and overrelease it within exosomes, effects suppressed by calcilytic NPS 2143⎯Further implications for Alzheimer’s therapy**

Anna Chiarini^1*^, Ubaldo Armato^1^, Emanuela Gardenal^1^, Li Gui^2^, Ilaria Dal Prà^1*^

## ^1^Human Histology & Embryology Unit, Medical School, University of Verona, Verona, Venetia, Italy

^2^Department of Neurology, Southwest Hospital, Third Military Medical University, Chongqing, China

1. **Materials and Methods**

# Human adult astrocytes cultures

Untransformed human adult astrocytes were isolated from anonymized surgical fragments of normal adult human temporal cortex (brain trauma leftovers) provided by several Neurosurgery Units after obtaining written informed consent from all the patients and/or their next-of-kin. Experimental use of isolated astrocytes was approved by the Ethical Committee of Verona’s University-Hospital Integrated Co. Prog. No. CE118CESC. All human cells experiments were performed in accordance with the relevant guidelines and regulations of Verona’s University-Hospital Integrated Company. Cultures of astrocytes were set up, as previously described [Armato et al. 2013], in a medium consisting of 89% (v/v) of a 1:1 mixture of DMEM and F-12 medium (Life Technologies Italia, Monza, Italy), 10% (v/v) heat-inactivated (at 56°C for 30 min) fetal bovine serum (FBS; Life Technologies Italia) and 1% (v/v) of a penicillin–streptomycin solution (Lonza Milano, Italy). Since usually astrocytes do not proliferate in the adult human brain, we employed them once they had reached mitotic quiescence.

- 1. **Experimental protocol**

At experimental “0 h”, some of such cultures served as untreated controls while others had 20 μM of fAβ_25–35_ added to their medium [Armato et al. 2013]. fAβ_25–35_ was used instead of fAβ_1-42_ because we assessed in parallel the release of endogenous fAβ_1-42_ as a control of the standard response of the astrocytes’ CaSR as we previously reported [Armato et al. 2013]. The CaSR allosteric antagonist (calcilytic) NPS 2143 hydrochloride (2-chloro-6-[(2R)-3-1,1-dimethyl-2-(2-naphtyl)ethylamino-2-hydroxy-propoxy]-benzonitrile hydrochloride; Tocris Bioscience, UK) [Nemeth and Goldman 2016] was dissolved in DMSO and next diluted in the growth medium at a final concentration of 100 nM. Starting at experimental “0 h” time and every 24-h thereafter astrocytes were first exposed for 30 min to NPS 2143 dissolved in fresh medium; thereafter, fresh (at 0.5 h) or the previously cell-conditioned (at 24.5 and 48.5 h) media were added again to the cultures. Cultures and cell-conditioned media were sampled at 24 hourly intervals. Phosphoramidon (10 μM; Sigma, Milan Italy), an inhibitor of thermolysin and other proteases, was added to the media at “0 h” experimental time.

- 1. **Aβ peptides**

Aβ_25-35_ (Bachem AG, Bubendorf, Switzerland) was dissolved at 1.5 mM in PBS. Fibrillogenesis by Aβ_25-35_ was fast (minutes) at room temperature (*rt*) and was checked *via* thioﬂavin-T tests before the experiments.

- 1. **Immunofluorescence**

Human astrocytes were fixed in 4% paraformaldehyde (30–min) and after 4 changes (5–min each) of PBS, the cells were permeabilized in 0.05% Triton X–100 (10–min). To saturate unspecific binding sites, cells were incubated for 45–min at *rt* with a blocking solution containing 2% FBS, 2% BSA and 0.02% Triton X-100 in PBS. Samples were then incubated overnight at 4°C with these primary antibodies: anti-human Tau mouse monoclonal antibody (HT7) (Thermo Scientific, Fremont, CA, USA) (5.0 μg L^-1^) diluted in blocking solution. After 3 washes with PBS, the cells were incubated for 1–h at *rt* in the dark with specific secondary antibodies (1.0 μg mL^−1^) conjugated with Alexa Fluor-488 (Molecular Probes, Invitrogen Corporation, Carlsbad, CA, USA). The incubation with the secondary antibody was followed by a 10–min incubation with 1.0 μg mL^−1^ of 4′,6-diamidino-2-phenylindole di-hydrochloride (DAPI, Sigma). Coverslips were mounted in anti-bleaching medium (Dabco, Sigma) in 50% glycerol. The negative control procedure omitted the primary antibody.

- 1. **Western immunoblotting (WB)**

Control and treated adult human astrocytes were scraped into cold PBS, sedimented at 200 x *g* for 10 min, and homogenized in T–PER™ tissue protein extraction reagent (Thermo Scientific, Rockford, USA) containing a complete EDTA–free protease inhibitor cocktail (Roche, Milan). The protein contents of the samples were assayed according to Bradford using BSA as standard. Equal amounts (10–30 mg) of protein from the samples were heat–denatured for 10 min at 70°C in an appropriate volume of 1X NuPAGE LDS Sample Buffer supplemented with 1X NuPAGE Reducing Agent (Life Technologies Italia). The samples and 2.0 µL of Tau Protein Ladder (Sigma Aldrich, Italy) were next loaded on NuPAGE Novex 4–12% Bis–Tris polyacrylamide gel (Life Technologies Italia). After electrophoresis in NuPAGE MOPS SDS Running Buffers using the Xcell SureLock™ Mini–Cell (Life Technologies Italia) (50–min runtime at 200 V constant), proteins were blotted onto nitrocellulose membranes (0.2 µm) by means of iBlot^TM^ 2 Dry Blotting System (Life Technologies Italia). The membranes were probed with: *(i)* anti-human Tau mouse monoclonal antibody (HT7; Thermo Scientific, USA) diluted at 1.0 μg mL^–1^ according to the seller’s documentation; *(ii)* anti-phospho-Ser^9^GSK-3β mouse monoclonal antibody (2D3; Merck-Millipore, Germany) before use it was diluted at 2.0 μg mL^–1^ according to the seller’s documentation; *(iii)* anti-phospho-Tyr^216^GSK-3β mouse monoclonal antibody (5G-2F; Merck-Millipore) which before use was diluted at 2.0 μg mL^–1^ according to the seller’s documentation; *(iv)* anti-total GSK-3 mouse monoclonal antibody (4G-1E; Merck-Millipore) before use it was diluted at 2.0 μg mL^–1^ according to the seller’s documentation; *(v)* goat polyclonal antibody anti-lamin B (Santa Cruz Biotechnology) used at 1.0 μg mL^–1^ to assess the loading controls. Alkaline phosphatase-conjugated goat anti-mouse IgG antibody was applied as secondary antibody (diluted 1:1000; Life Technologies). The integrated intensities of the bands specific for each protein of interest were assessed using the Sigmagel™ software package (Jandel Corp., Erkrath, Germany).

- 1. **Isolation of phospho (p)-Tau**

PhosphoCruzAgarose™ (Santa Cruz Biotechnology) was used according to the manufacturer's instructions. Briefly, 300 μg whole lysate proteins were diluted up to 1.0 mL with 50 mM MES, 1.0 M NaCl, 0.25% CHAPS pH 6.6 (binding/washing buffer), and reacted with PhosphoCruzAgarose for 90 min at 4˚C under swelling. Following 3 washes with binding/washing buffer, the phosphoproteins were eluted from PhosphoCruzAgarose in 100 mM ammonium bicarbonate, 0.25% CHAPS pH 9.0 (elution buffer) and assessed by western blot analysis using anti-human Tau mouse monoclonal antibody (HT7; Thermo Scientific).

- 1. **Total exosome isolation from astrocytes-conditioned growth media**

To ensure that isolated exosomes originate from human astrocytes-conditioned growth media, the cells were cultured with exosome depleted FBS. To this aim, FBS was centrifuged at 100.000 x g for 90 min twice to remove any contaminating bovine exosomes and then was added to the cell culture medium. Astrocytes-conditioned media were collected at 24 hourly intervals and total exosomal fractions were prepared by means of the Total Exosome Isolation Reagent for cell culture media (provided by Life Technologies Italia) according to the protocol recommended by the supplier's instructions.

- 1. **Enzyme-linked immunosorbant assays (ELISAs) of secreted human total Tau and p-Tau in astrocytes-conditioned growth media samples and in total exosome fractions**

Quantifications of human total Tau and p-Tau released into cell-conditioned growth media samples and contained in the total exosome fractions were carried out by means of the specific HumanTau ELISA Kit, (Life Technologies Italia) and Human P-Tau ELISA Kit (BlueGene Biotech, China). Briefly, the astrocytes’ conditioned media samples were added with a protease inhibitor cocktail (Roche) and centrifuged for 10 min at 13,000 rpm to remove any cellular debris. Supernatants were tested in triplicate according to the manufacturer’s protocol. Total exosomes were isolated from cell cultures, re-suspended in PBS as previously described and tested according to the manufacturer’s protocol.

#### Statistical analysis

The data were analyzed using Sigma Stat 3.5™ Advisory Statistics for Scientists (Systat Software). For immunoblotting, bands’ densitometric data were normalized to matching loading control (lamin B1) bands and next analyzed by one–way ANOVA. When the ANOVA’s upshot was significant (*P* < 0.05), *post hoc* Bonferroni’s test was used for comparisons *vs*. 0-h (untreated) control values and for multiple comparisons. Null hypotheses were rejected when *P >* 0.05.

**References**

Armato, U., Chiarini, A., Chakravarthy, B., Chioffi, F., Pacchiana, R., Colarusso, E., et al. (2013). Calcium-sensing receptor antagonist (calcilytic] NPS 2143 specifically blocks the increased secretion of endogenous Aβ42 prompted by exogenous fibrillary or soluble Aβ25-35 in human cortical astrocytes and neurons-therapeutic relevance to Alzheimer’s disease. *Biochim Biophys Acta* 1832, 1634–1652. doi: 10.1016/j.bbadis.2013.04.020

Nemeth, E. F., Goodman, W. G. (2016) Calcimimetic and calcilytic drugs: Feats, flops, and futures. *Calcif. Tissue Int.* 98(4), 341-358. doi: 10.1007/s00223-015-0052-z
